# Efficacy of high-vision transnasal endoscopy using texture and colour enhancement imaging and narrow-band imaging to evaluate gastritis: a randomized controlled trial

**DOI:** 10.1080/07853890.2022.2063372

**Published:** 2022-04-20

**Authors:** Mitsushige Sugimoto, Yusuke Kawai, Yuko Morino, Mariko Hamada, Eri Iwata, Ryota Niikura, Naoyoshi Nagata, Yohei Koyama, Masakatsu Fukuzawa, Takao Itoi, Takashi Kawai

**Affiliations:** a Department of Gastroenterological Endoscopy, Tokyo Medical University Hospital, Tokyo, Japan; b Tokyo University of Pharmacy and Life Sciences, Tokyo, Japan; c Department of Gastroenterology, Tokyo Medical University Hospital, Tokyo, Japan

**Keywords:** Texture and colour enhancement imaging, narrow-band imaging, transnasal endoscopy, Kyoto classification of gastritis, *H. pylori*

## Abstract

**Background:**

A new image-enhanced endoscopy method called texture and colour enhancement imaging (TXI) enhances brightness, surface irregularities, and subtle colour changes in endoscopic images. However, it is unclear whether TXI and narrow-band imaging (NBI) with third-generation high-vision transnasal ultrathin endoscopy are advantageous over white-light imaging (WLI) for detecting atrophy, intestinal metaplasia, map-like redness and gastric cancer. We investigated to compare the endoscopic efficacy for evaluation of gastritis between TXI and NBI with high-vision transnasal endoscopy and clarified the endoscopic efficacy of TXI and NBI compared to WLI.

**Methods:**

We enrolled 60 patients who underwent high-vision transnasal endoscopy as part of a health check-up from March to November 2021 and randomized patients into two groups (the WLI-NBI group and the WLI-TXI group) using the minimization method based on *Helicobacter pylori* infection status, age and sex. Colour differences determined using the International Commission on Illumination 1976 (L∗, a∗, b∗) colour space was compared between WLI and TXI or NBI.

**Results:**

No significant differences were observed in colour differences surrounding atrophy, intestinal metaplasia and map-like redness between NBI and TXI (*p* = .553, .057 and .703, respectively). Endoscopic scores based on the Kyoto classification of gastritis for atrophy, intestinal metaplasia, and map-like redness were similar between WLI and TXI. In contrast, NBI identified intestinal metaplasia at a significantly greater rate than WLI (*p* = .018). Further, colour differences surrounding atrophy and intestinal metaplasia on TXI and NBI were significantly greater than those on WLI (atrophy: TXI vs WLI *p* = .003, NBI vs WLI *p* < .001; intestinal metaplasia: TXI vs WLI *p* = .016, NBI vs WLI *p* < .001). However, TXI and NBI were not advantageous over WLI for detecting map-like redness.

**Conclusion:**

Third-generation high-vision transnasal ultrathin endoscopy using TXI and/or NBI is useful for detecting atrophic borders and intestinal metaplasia.

**Key Messages**
- High-vision transnasal endoscopy using TXI or NBI is useful for diagnosing and detecting atrophy and intestinal metaplasia.
- TXI and NBI increase colour differences surrounding atrophy and intestinal metaplasia, thereby increasing diagnostic efficiency to improve risk stratification for gastric cancer.
- The image quality and detection rate have improved markedly with the latest ultrathin high-vision transnasal endoscopes.

## 1. Introduction

Gastric cancer that arises from long-term *Helicobacter pylori* infection is a major concern around the world. Although gastric cancer is generally caused by a multifactorial and multistep process, severe atrophy and intestinal metaplasia are well-known risk factors for gastric cancer [1,2]. The Kyoto classification of gastritis used in Japan for evaluating endoscopic characteristics of gastritis provides a system for grading risk factors for gastric cancer [2,3]. Based on this system, patients are divided into three groups: those who are *H. pylori*-negative (no gastritis), those with current infection (active gastritis), and those who have previously been infected (inactive gastritis), and are assessed for gastric cancer risk by scoring five parameters, namely atrophy, intestinal metaplasia, enlarged folds, nodularity, and diffuse redness [2,3]. Map-like redness is characteristic endoscopic findings in inactive gastritis [4] and has good specificity for past *H. pylori* infection [specificity 0.99 (95%CI: 0.95–1.00)] [4]. In addition, the map-like redness that is considered to represent histologic intestinal metaplasia are independent risk factors for newly detected gastric cancer [5,6]. In fact, it was significantly more frequent in patients with newly detected gastric cancer after eradication than in those without gastric cancer using white-light imaging (WLI; 61.5% vs. 37.7%, *p* = .001) and linked colour imaging (LCI; 78.0% vs. 45.9%, *p* < .0001) [6]. Therefore, it is important to correctly evaluate not only gastric atrophy and intestinal metaplasia, but also map-like redness in patients at high risk of gastric cancer. However, the current routine endoscopic evaluation method, WLI, is ineffective for detecting the presence of these risk factors.

Recent developments in endoscopic instrumentation and image-enhancement techniques, known together as image-enhanced endoscopy (IEE), including narrow-band imaging (NBI), blue laser imaging, and LCI with or without magnification, have improved the detection rate of gastric cancer and intestinal metaplasia [6–10]. According to the MAPS II guideline, an official statement from the European Society of Gastrointestinal Endoscopy, high-definition endoscopy with IEE is more effective for detecting atrophy and intestinal metaplasia than high definition WLI alone [11]. Texture and colour enhancement imaging (TXI) is a new IEE modality that utilizes Retinex theory-based image processing technology. TXI specifically enhances three imaging factors in WLI (texture, brightness, and colour) to clearly define subtle tissue differences. TXI can selectively enhance brightness in dark areas of an endoscopic image and subtle tissue differences such as slight morphological or colour changes while simultaneously preventing over-enhancement [12]. Therefore, although combining WLI with TXI is expected to increase the detection rate of endoscopic atrophy and intestinal metaplasia, efficacy of TXI for detection of gastrointestinal diseases is unclear.

Transnasal endoscopy has become a popular medical screening test in Japan because it is relatively pain-free for the patient. To date, no reports have compared the effectiveness of WLI and TXI for objectively evaluating risk factors for gastric cancer (e.g. atrophy, intestinal metaplasia, and map-like redness) using high-vision ultrathin endoscopy. An increase in health check-ups is leading to a parallel rise in the conduct of transnasal endoscopy tests, making it prudent to evaluate the usefulness of these tests.

Here, we investigated to compare the endoscopic efficacy for evaluation of gastritis between TXI and NBI with high-vision third-generation transnasal ultrathin endoscopy and clarified whether endoscopy with TXI or NBI improves the visibility of gastric cancer risk factors identified in the Kyoto classification of gastritis compared with standard WLI.

## 2. Methods

### 2.1. Study design and patients

**Figure 1.**
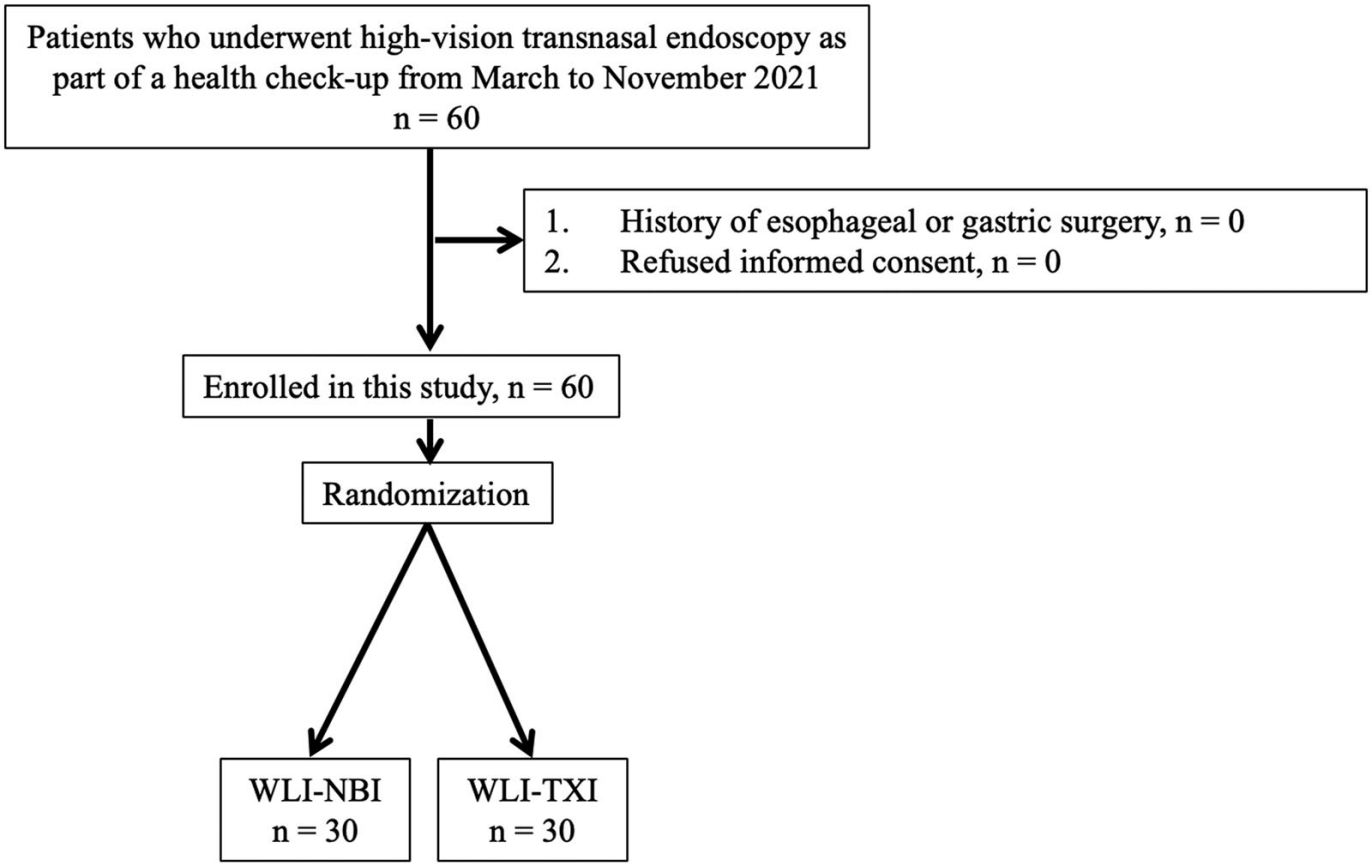
Workflow for patient enrolment in this study.

This was a prospective randomized controlled trial conducted at the Tokyo Medical University Hospital to investigate the efficacy of TXI and NBI with third-generation high-vision ultrathin transnasal endoscopy for evaluating gastritis. We enrolled 60 patients aged ≥20 years who underwent high-vision transnasal endoscopy as part of a health check-up between March and November 2021. Exclusion criteria were patients with a history of gastric surgery. Written informed consent was obtained from all patients. In this study, using the minimization method based on *H. pylori* infection status (Negative, Current and Eradicated), age and sex (male and sex), we randomized patients into two groups (the WLI-NBI group evaluated gastritis by both WLI and NBI, *n* = 30; and the WLI-TXI group evaluated by both WLI and TXI, *n* = 30; Figure 1). Endoscopic evaluation was performed twice in succession using the two selected methods. We compared colour differences of atrophy, intestinal metaplasia and map-line redness between NBI score of the WLI-NBI group and TXI score of the WLI-TXI group as the primary endpoint and compared colour differences of atrophy, intestinal metaplasia and map-line redness between WLI and TXI or NBI, as the secondly endpoint.

The study protocol adhered to the ethical principles of the Declaration of Helsinki and was approved by the institutional review board of Tokyo Medical University (T2020-0389). This trial was registered in the University Hospital Medical Information Network (UMIN000044149).

### 2.2. Endoscopy and severity of gastritis

Endoscopy was performed using a third-generation high-vision GIF-1200N transnasal endoscope with the EVIS X1 system (Olympus Co., Tokyo, Japan). Initially, before transnasal endoscopy, patients were provided a solution containing dimethylpolysiloxane and pronase as premedication to improve gastrointestinal mucosal visibility, and also given a naphazoline nitrate spray to both nostrils to prevent nasal edoema and haemorrhage. Next, local anaesthesia with 2% lidocaine gel was provided to the nostrils and a pre-treatment nasal catheter coated with lidocaine gel was gently inserted into either nostril so that they would not feel pain until the beginning of the endoscopic examination.

The severity of gastritis was scored according to the Kyoto classification of gastritis and the Kimura–Takemoto classification [3,13]. Endoscopic findings are reliable in the diagnosis of an atrophic border and the endoscopic features of mucosal atrophy are characterized by a discoloured mucosa and visible capillary network in the atrophic area [14]. Intestinal metaplasia is defined as multiple ashen nodular or cobblestone-like lesions on atrophic mucosa observed using WLI and TXI. Villous appearance, whitish mucosa, and rough mucosal surface are helpful indicators for the endoscopic diagnosis of intestinal metaplasia. A white opaque substance on the surface epithelium and light blue crest on the mucosal epithelial rim visualized using NBI are associated with intestinal metaplasia. Map-like redness is defined as reddish depressed areas of various shape and sizes in the atrophic area using WLI and TXI [14]. In the Kyoto classification, the total score is calculated by summing the scores for five parameters, namely atrophy (Kimura–Takemoto classification CI: Kyoto A0, CII–CIII: Kyoto A1, OI–OIII: Kyoto A2), intestinal metaplasia (none: IM0, within antrum: IM1, up to corpus: IM2), hypertrophy of gastric folds (negative: H0, positive: H1), nodularity (negative: N0, positive: N1), and diffuse redness (negative: DR0, mild: DR1, moderate-severe: DR2) [2,15–17]. Two expert endoscopists independently evaluated the severity of gastritis using WBI, NBI and TXI after endoscopy. During endoscopy, more than 100 pictures were taken by an expert endoscopist (KT). When scores assigned by the two endoscopists differed, consensus was reached by reviewing the pictures.

### 2.3. Measurement of colours

**Figure 2.**
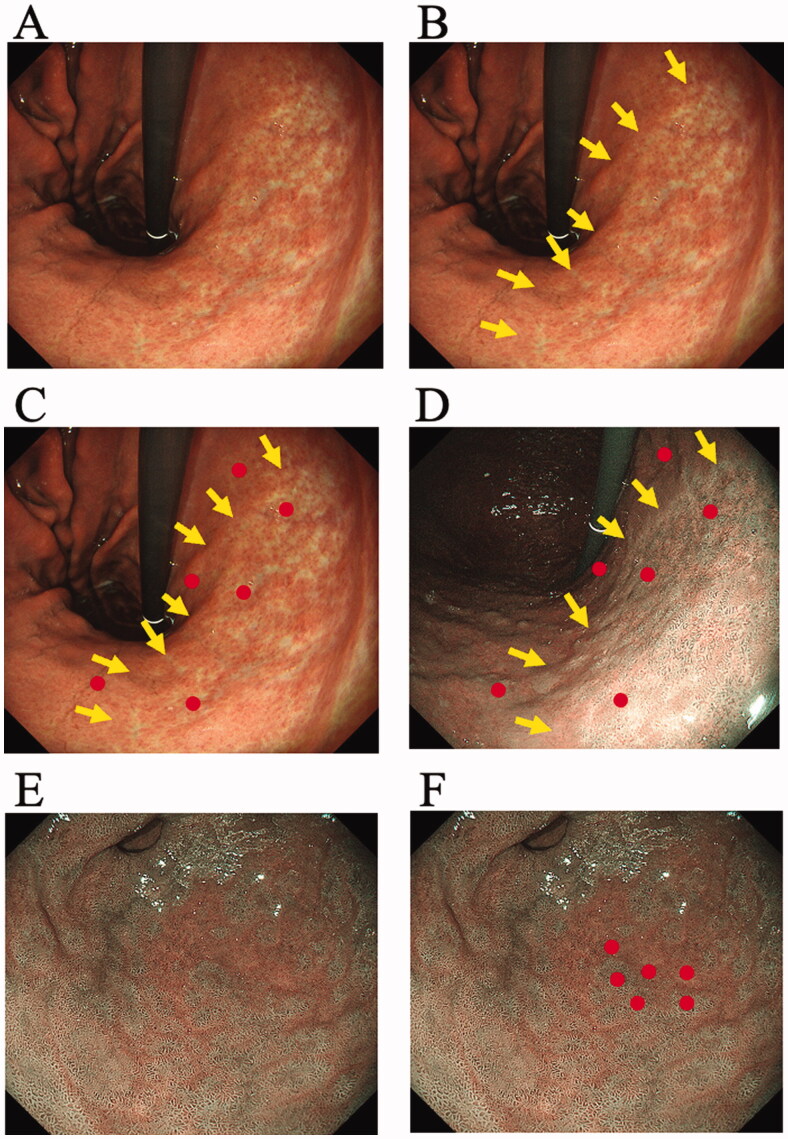
Measurement of colour differences of atrophy, intestinal metaplasia and map-like redness using the endoscopic images. Each sample area at the atrophic border, intestinal metaplasia and map-like redness was imaged by between white-light imaging (WLI) and narrow-band imaging (NBI) and between WLI and texture and colour enhancement imaging (TXI) with the similar composition. (A) The atrophic border is seen in lesser curvature of gastric body. (B) Two expert endoscopists independently set a sample area of the atrophic mucosa by WLI image and delineated the margin of atrophic border on the WLI image (Arrows). (C) The endoscopist manually annotated three points 3–5 mm inside and outside the atrophic border (Circles). (D) The inside and outside points were similarly annotated on the image of NBI with the similar angle and distance. We set three pairs of sample areas and calculated the colour difference between atrophic and non-atrophic mucosal areas with each image of WLI and NBI. (E) The intestinal metaplasia is seen in antrum on the NBI image. (F) The endoscopist set a sample area of the intestinal metaplasia by NBI image and manually annotated three points inside and outside the intestinal metaplasia (Circles). We set three pairs of sample areas and calculated the colour difference between intestinal metaplasia and non- intestinal metaplasia with each image of WLI and NBI.

**Figure 3.**
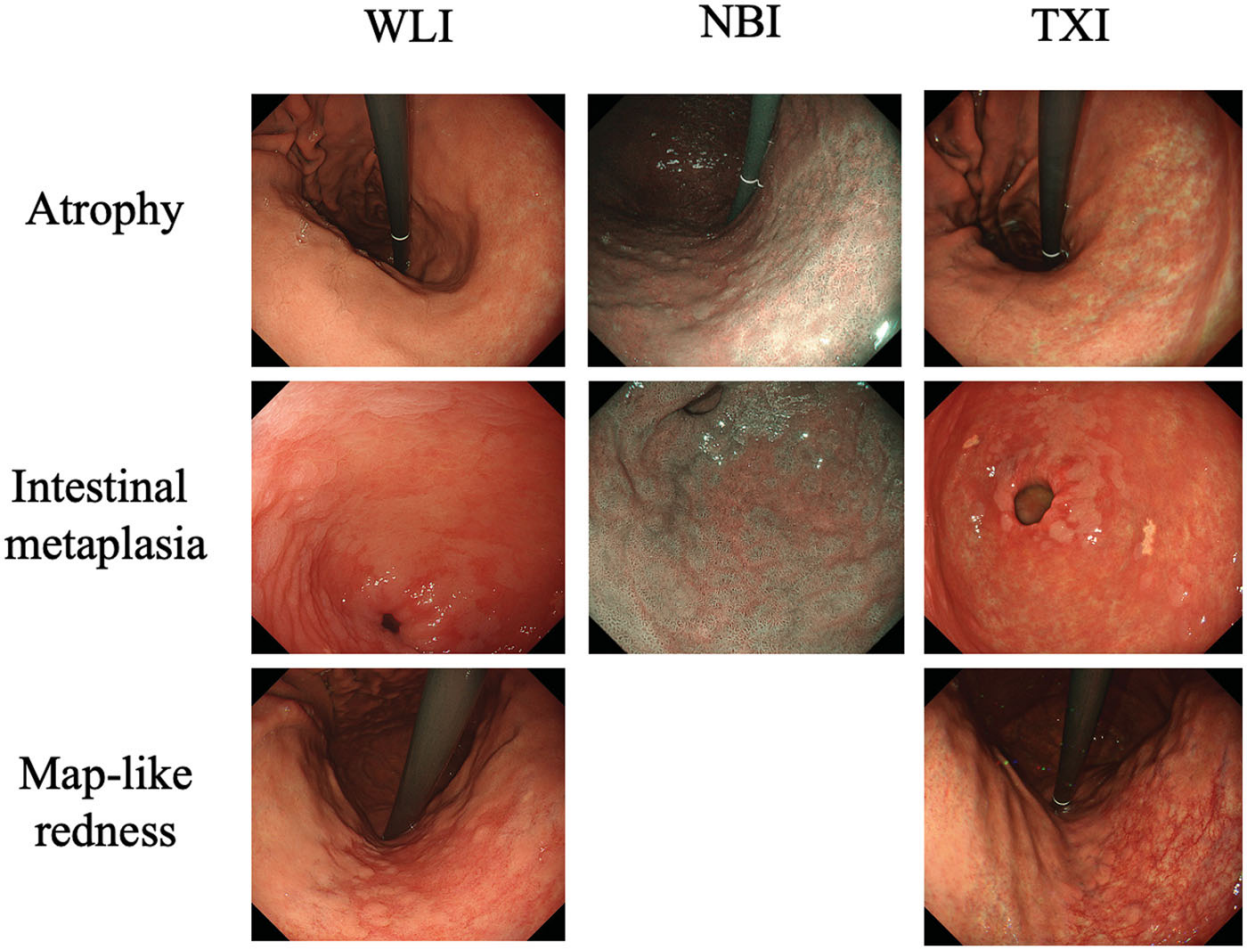
Typical endoscopic images of atrophy, intestinal metaplasia and map-like redness taken using a third-generation GIF-1200N ultrathin endoscope by white-light imaging (WLI), narrow-band imaging (NBI) and texture and colour enhancement imaging (TXI).

Colour differences surrounding atrophic borders, intestinal metaplasia and map-like redness were measured and compared between NBI score of the WLI-NBI group and TXI score of the WLI-TXI group and between WLI and NBI or TXI (Figure 2 and 3) [10]. We randomly set three pairs at atrophic border, intestinal metaplasia and map-like redness and calculated the colour difference in each patient. Colour differences were calculated using the International Commission on Illumination (CIE) 1976 (L*, a*, b*) colour space [18,19], a three-dimensional model composed of a black-white axis (L*, brightness), a red-green axis (a*, red-green component), and a yellow-blue axis (b*, yellow-blue component). A colour difference was defined as Δ*E*, which expresses the distance between two points in the colour space. Δ*E* was calculated using the following formula: {(ΔL*)^2^+(Δa*)^2^+(Δb*) [2]}^1/2^, where ΔL*, Δa*, and Δb* are differences in the L*, a*, and b* values, respectively, between regions with and without atrophy, intestinal metaplasia and map-like redness. Each ΔL*, Δa*, and Δb* value was determined by a computer operator who was blinded to clinical information using Adobe Photoshop, version 22.5.1 (Adobe KK, Tokyo, Japan).

### 2.4. *Helicobacter pylori* infection

*Helicobacter pylori* infection was diagnosed in all patients using the rapid urease test (Helicocheck^®^; Institute of Immunology, Co., Ltd., Tochigi, Japan), and an anti-*H. pylori* IgG serological test. Patients were diagnosed as *H. pylori* infection-positive if they received positive results in at least one of the two tests. Patients who obtained negative results in both tests for *H. pylori* infection and had no signs of endoscopic atrophy or intestinal metaplasia were diagnosed as *H. pylori*-negative.

### 2.5. Statistical analysis

When we set 0.75 of effect size, 0.05 of alpha error, 0.80 of sample power (1-beta), 1 of allocation ratio for differences between two independent means (two groups) at two tails by the *t-*test, sample number of each group is required 29 patients [G*Power software (ver. 3.1.9.6), Heinrich-Heine-Universität Düsseldorf, Düsseldorf, Germany]. Therefore, we enrolled 60 patients (each group: 30 patients) in this study.

Parameters including age, height, body weight, Kyoto classification score and F-scale questionnaire score are expressed as mean ± standard deviation (SD). Categorical variables for WLI, NBI and TXI are summarized as *n* (%) and were compared using *χ*^2^ tests. Statistically significant differences in mean Kyoto classification scores, F-scale questionnaire scores and mean Δ*E* between NBI score of the WLI-NBI group and TXI score of the WLI-TXI group and between WLI and NBI or TXI were determined using Student’s *t*-test. A *p*-value <.05 was considered statistically significant, and all *p*-values were two-sided. All statistical analyses were performed using the statistical analysis software SPSS, version 27.0 (IBM Japan, Tokyo, Japan).

## 3. Results

### 3.1. Patient characteristics

We enrolled a total of 60 patients who underwent transnasal endoscopy as part of a health check-up (Figure 1). Because no patient had a history of surgery, we randomized all 60 patients into two groups (WLI-NBI and WLI-TXI groups). Data from all 60 patients were evaluated.

The mean age was 72.7 ± 7.7 years and 56.7% of patients were male (Table 1). *H. pylori* infection had been eradicated in 86.7% (52/60) of patients and *H. pylori*-negative patients comprised 11.7% (7/60). Apart from a significant difference in the rate of hyperlipidaemia and hypertension between WLI-NBI and WLI-TXI groups, no significant differences were observed in any characteristics, drugs, or severity of abdominal symptoms as evaluated by the F-scale between the two groups (Table 1).

**Table 1.** Characteristics of patients.

	All patients (*n* = 60)	WLI-NBI (*n* = 30)	WLI-TXI (*n* = 30)	*p* value
Age (years ± SD)	72.7 ± 7.7	73.0 ± 7.6	72.3 ± 7.8	.726
Sex [male, *n* (%)]	34 (56.7%)	16 (53.3%)	18 (60.0%)	.602
Height (cm ± SD)	160.5 ± 16.2	161.5 ± 9.7	159.4 ± 20.9	.618
Body weight (kg ± SD)	59.8 ± 11.0	59.6 ± 11.7	59.9 ± 10.4	.917
*H. pylori* infection, Negative/Current/Eradicated [*n*/*n*/*n*]	7/1/52	3/1/26	4/0/26	.565
Smoking, Never/Current/Past [*n*/*n*/*n*]	39/1/20	19/0/11	20/1/9	.532
Alcohol [*n* (%)]	38 (63.3%)	19 (63.3%)	19 (63.3%)	1.000
Diseases				
Peptic ulcer [*n* (%)]	5 (8.3%)	3 (10.0%)	2 (6.7%)	.640
Gastric cancer [*n* (%)]	3 (5.0%)	2 (6.7%)	1 (3.3%)	.544
Cancer (others) [*n* (%)]	15 (25.0%)	5 (16.7%)	10 (33.3%)	.136
Hyperlipidaemia [*n* (%)]	22 (36.7%)	15 (50.0%)	7 (23.3%)	.032
Hypertension [*n* (%)]	30 (50.0%)	19 (63.3%)	11 (36.7%)	.039
Diabetes mellites [*n* (%)]	11 (18.3%)	7 (23.3%)	4 (13.3%)	.317
Drugs				
PPI [*n* (%)]	18 (30.0%)	9 (30.0%)	9 (30.0%)	1.000
NSAID [*n* (%)]	0 (0%)	0 (0%)	0 (0%)	
Antiplatelet drug [*n* (%)]	17 (28.3%)	8 (26.7%)	9 (30.0%)	.774
Anticoagulant [*n* (%)]	5 (8.3%)	3 (10.0%)	2 (6.7%)	.640
Bisphosphonate	2 (3.3%)	0 (0%)	2 (6.7%)	.150
F-scale questionnaire				
Acid-related symptom score	2.5 ± 3.4	2.1 ± 2.7	2.9 ± 4.0	.349
Dysmotility-related symptom score	2.2 ± 2.7	1.6 ± 2.2	2.7 ± 3.1	.120
*F*-scale total score	4.6 ± 5.8	3.7 ± 4.8	5.6 ± 6.7	.196

Abbreviations: *H. pylori*: *Helicobacter pylori*; NBI: narrow-band imaging; SD: standard deviation; TXI: texture and colour enhancement imaging; WLI: white-light imaging.

Abdominal symptoms were evaluated using the F-scale questionnaires [31,32]. A total score of ≥8 indicates probable GERD. The 12 items are classified into 2 domains, a reflux-related symptom domain and an acid-related dysmotility symptom domain.

### 3.2. Colour differences in endoscopic atrophy, intestinal metaplasia and map-like redness between NBI score and TXI score

The colour difference surrounding atrophic borders was 19.3 ± 8.0 in NBI and 20.8 ± 9.7 in TXI; the colour difference in NBI and WLI was similar (*p* = .553, Table 2). The colour difference surrounding intestinal metaplasia in NBI was 13.5 ± 4.7, which was no significant difference with TXI (10.9 ± 3.8, *p* = .057). No significant differences were observed in Δ*E* surrounding map-like redness between NBI and TXI (*p* = .703).

**Table 2.** Colour differences in endoscopic atrophy, intestinal metaplasia and map-like redness between NBI score and TXI score.

Characteristic		WLI-NBI	WLI-TXI	*p* value
		NBI	TXI	
Atrophy	ΔL*	13.1 ± 12.1	12.0 ± 14.1	.750
	Δa*	6.1 ± 6.1	–9.0 ± 9.0	<.001
	Δb*	2.6 ± 5.3	0.1 ± 4.8	.071
	ΔE*	19.3 ± 8.0	20.8 ± 9.7	.553
Intestinal metaplasia	ΔL*	5.5 ± 6.4	3.2 ± 4.6	.194
	Δa*	–10.1 ± 3.7	3.3 ± 8.8	<.001
	Δb*	–2.0 ± 4.0	1.4 ± 4.0	.010
	ΔE*	13.5 ± 4.7	10.9 ± 3.8	.057
Map-like redness	ΔL*	–3.7 ± 2.5	–9.4 ± 3.4	.032
	Δa*	–9.0 ± 10.4	8.3 ± 6.2	.010
	Δb*	1.7 ± 5.7	1.4 ± 3.3	.933
	ΔE*	11.7 ± 9.3	13.5 ± 5.6	.703

Abbreviations: *H. pylori*: *Helicobacter pylori*; LCI: linked colour imaging; WLI: white-light imaging; ΔL*: change in brightness; Δa*: change in red-green component; Δb*: change in yellow-blue component; Δ*E*: colour difference.

### 3.3. Severity of endoscopic gastritis assessed by various IEE methods

The severity of gastritis based on the degree of atrophy, intestinal metaplasia, enlarged folds, nodular gastritis and diffuse redness was similar between WLI and TXI in the WLI-TXI group (Tables 3 and 4). In contrast, although the severity of atrophy, enlarged folds, nodular gastritis and diffuse redness was similar between WLI and NBI in the WLI-NBI group, the distribution of the severity of intestinal metaplasia significantly differed between WLI and NBI (*p* = .018), with the mean score for intestinal metaplasia in NBI being significantly higher than that in WLI (1.50 ± 0.57 vs. 0.90 ± 0.92, *p* = .007; Tables 3 and 4).

**Table 3.** Severity of endoscopic gastritis among different characteristics or categories of *H. pylori* infection.

Category/characteristic		WLI-NBI		*p*-value	WLI-TXI		*p*-value
		WLI	NBI		WLI	TXI	
Kimura–Takemoto classification	C-O–C-II	9 (30.0%)	6 (20.0%)	0.140	7 (23.3%)	6 (20.0%)	0.375
	C-III–O-I	15 (50.0%)	22 (73.3%)		16 (53.3%)	12 (40.0%)	
	O-II–O-III	6 (20.0%)	2 (6.6%)		7 (23.3%)	12 (40.0%)	
Kyoto classification of gastritis							
Atrophy	A0	3 (10.0%)	1 (3.3%)	0.491	5 (16.7%)	5 (16.7%)	0.743
	A1	10 (33.3%)	13 (45.3%)		5 (16.7%)	3 (10.0%)	
	A2	17 (56.7%)	16 (53.3%)		20 (66.7%)	22 (73.3%)	
Intestinal metaplasia	IM0	14 (46.7%)	4 (13.3%)	**0.018**	13 (43.3%)	11 (36.7%)	0.538
	IM1	5 (16.7%)	7 (23.3%)		5 (16.7%)	3 (16.7%)	
	IM2	11 (36.7%)	19 (63.3%)		12 (40.0%)	16 (53.3%)	
Enlarged fold	H0	29 (96.7%)	30 (100%)	0.313	30 (100%)	30 (100%)	–
	H1	1 (3.3%)	0 (0%)		0 (0%)	0 (0%)	
Nodular gastritis	N0	30 (100%)	30 (100%)	–	30 (100%)	30 (100%)	–
	N1	0 (0%)	0 (0%)		0 (0%)	0 (0%)	
Diffuse redness	DR0	28 (93.3%)	–	–	26 (86.7%)	27 (90.0%)	0.151
	DR1	1 (3.3%)	–		4 (13.3%)	1 (3.3%)	
	DR2	1 (3.3%)	–		0 (0.0%)	2 (6.7%)	
Other findings							
Xanthoma [*n* (%)]		6 (20.0%)	6 (20.0%)	1	6 (20.0%)	6 (20.0%)	1
Multiple white and flat elevated lesions [*n* (%)]		4 (13.3%)	9 (30.0%)	0.117	2 (6.6%)	2 (6.6%)	1
Map-like redness [*n* (%)]		4 (13.3%)	5 (16.7%)	0.718	9 (30.0%)	10 (33.3%)	0.781

Abbreviations: NBI: narrow-band imaging; SD: standard deviation; TXI: texture and colour enhancement imaging; WLI: white-light imaging.

**Table 4.** Endoscopic severity of gastritis according to the Kyoto classification of gastritis.

Characteristic	WLI-NBI		*p*-value	WLI-TXI		*p-*value
	WLI	NBI		WLI	TXI	
Atrophy	1.47 ± 0.68	1.50 ± 0.57	0.837	1.50 ± 0.78	1.57 ± 0.77	0.740
Intestinal metaplasia	0.90 ± 0.92	1.50 ± 0.57	**0.007**	0.97 ± 0.93	1.17 ± 0.95	0.413
Enlarged fold	0.03 ± 0.00	0.00 ± 0.00	–	0.00 ± 0.00	0.00 ± 0.00	–
Nodular gastritis	0.00 ± 0.00	0.00 ± 0.00	0.321	0.00 ± 0.00	0.00 ± 0.00	–
Diffuse redness	0.10 ± 0.40	–	0.179	0.13 ± 0.35	0.17 ± 0.53	0.774
Total score	2.37 ± 1.65	2.97 ± 1.25	0.117	2.63 ± 1.71	2.80 ± 1.85	0.718

Data show mean ± standard deviation.

Abbreviations: NBI: narrow-band imaging; SD: standard deviation; TXI: texture and colour enhancement imaging; WLI: white-light imaging. Bold meant data with *p* < 0.05 with significant different.

The rate of detection of xanthoma, multiple white and flat elevated lesions and map-like redness were similar between WLI and NBI in the WLI-NBI group and between WLI and TXI in the WLI-TXI group (Table 3).

### 3.4. Colour differences in atrophy, intestinal metaplasia and map-like redness between WLI and NBI or TXI

The colour difference surrounding atrophic borders was 14.5 ± 5.9 in WLI and 19.3 ± 8.0 in NBI; the difference in NBI was significantly greater than that in WLI (*p* = .016, Table 5). The colour difference surrounding intestinal metaplasia in NBI was 13.5 ± 4.7, which was significantly greater than that in WLI (6.6 ± 3.2, *p* < .001). Significant differences were also observed along the red-green axis (Δa*) surrounding atrophy and both the black-white (ΔL*) and red-green axes surrounding intestinal metaplasia between WLI and NBI in the WLI-NBI group. In contrast, no significant differences were observed in ΔL*, Δa*, Δb*, or Δ*E* surrounding map-like redness between WLI and NBI in the WLI-NBI group.

**Table 5.** Colour differences in atrophy, intestinal metaplasia and map-like redness between WLI and NBI or TXI.

Characteristic		WLI-NBI		*p*-value	WLI-TXI		*p*-value
		WLI	NBI		WLI	TXI	
Atrophy	ΔL*	8.1 ± 10.1	13.1 ± 12.1	.102	4.3 ± 13.1	12.0 ± 14.1	.034
	Δa*	–5.8 ± 5.3	6.1 ± 6.1	<.001	–5.4 ± 5.4	–9.0 ± 9.0	.038
	Δb*	0.7 ± 4.1	2.6 ± 5.3	.126	–1.3 ± 2.4	0.1 ± 4.8	.108
	ΔE*	14.5 ± 5.9	19.3 ± 8.0	**.016**	14.0 ± 7.3	20.8 ± 9.7	**.003**
Intestinal metaplasia	ΔL*	0.8 ± 4.0	5.5 ± 6.4	.002	3.2 ± 3.6	3.2 ± 4.6	.500
	Δa*	0.8 ± 5.7	–10.1 ± 3.7	<.001	–1.5 ± 4.6	3.3 ± 8.8	.004
	Δb*	0.3 ± 2.5	–2.0 ± 4.0	.057	–0.7 ± 2.5	1.4 ± 4.0	.018
	ΔE*	6.6 ± 3.2	13.5 ± 4.7	**<.001**	6.5 ± 3.1	10.9 ± 3.8	**<.001**
Map-like redness	ΔL*	–3.7 ± 2.1	–3.7 ± 2.5	1.000	–9.0 ± 4.3	–9.4 ± 3.4	.769
	Δa*	4.0 ± 3.6	–9.0 ± 10.4	.249	8.3 ± 3.1	8.3 ± 6.2	1.000
	Δb*	0.0 ± 1.7	1.7 ± 5.7	.652	1.7 ± 2.4	1.4 ± 3.3	.842
	ΔE*	5.8 ± 3.7	11.7 ± 9.3	.214	13.1 ± 3.1	13.5 ± 5.6	.885

Abbreviations: *H. pylori*: *Helicobacter pylori*; LCI: linked colour imaging; WLI: white-light imaging; ΔL*: change in brightness; Δa*: change in red-green component; Δb*: change in yellow-blue component; Δ*E*: colour difference. Bold meant data with *p* < 0.05 with significant different.

The colour difference surrounding atrophic borders was 14.0 ± 7.3 in WLI and 20.8 ± 9.7 in TXI; the difference in TXI was significantly greater than that in WLI (*p* = .003, Table 5). The colour difference surrounding intestinal metaplasia in NBI was 10.9 ± 3.8, which was significantly greater than that in WLI (6.5 ± 3.1, *p* < .001). Significant differences were observed along both the black-white and red-green axes surrounding atrophy and both the red-green and yellow-blue axes (Δb*) surrounding intestinal metaplasia between WLI and TXI in the WLI-TXI group. In contrast, there were no significant differences in ΔL*, Δa*, Δb*, or Δ*E* surrounding map-like redness between WLI and TXI in the WLI-TXI group.

No significant colour differences were observed in WLI surrounding atrophy (*p* = .817) or intestinal metaplasia (*p* = .909) between WLI-NBI and WLI-TXI groups (Table 5).

## 4. Discussion

We demonstrated that no significant differences were observed in colour differences based on the CIE 1976 (L*, a*, b*) colour space surrounding atrophic borders, intestinal metaplasia and map-like redness between NBI and TXI by third-generation high-vision transnasal ultrathin endoscopy and that TXI and NBI produced significantly greater colour differences surrounding atrophic borders and intestinal metaplasia than WLI. In particular, NBI significantly increased the detection rate of endoscopic intestinal metaplasia compared with WLI. Further, the severity of intestinal metaplasia according to the Kyoto classification of gastritis was significantly different between WLI and NBI. Therefore, combining high-vision ultrathin endoscopy with IEE may be useful for identifying patients at high risk of gastric cancer at health check-ups.

In a meta-analysis, compared with *H. pylori*-positive gastritis patients, those who had received eradication therapy had a significantly lower risk of gastric cancer, with a relative risk of 0.67 [95% confidence interval (CI), 0.47–0.96] for those with atrophic gastritis alone and 0.51 (95% CI, 0.36–0.73) for those who had undergone resection for gastric cancer [20]. This observation suggests that although eradication therapy has effectively reduced the risk of gastric cancer, surveillance of all patients who have received eradication is important for early diagnosis and to improve prognosis. Therefore, appropriate grading of the risk of gastric cancer is required to identify high-risk patients in whom regular surveillance should be of greater priority. The endoscopic severity of atrophy and intestinal metaplasia according with the Kyoto classification in patients with gastric cancer was significantly higher than that in subjects with gastritis alone [2]. Moreover, the MAPS II guideline states that patients with intestinal metaplasia, and with severe atrophic gastritis (OLGA/OLGIM stage III and IV) should be followed up with a high-quality endoscopy every 3 years [11]. Therefore, careful identification of patients with severe atrophy and intestinal metaplasia may be useful for grading those at high risk of gastric cancer.

Although WLI is currently the most common endoscopic technique for the stomach, TXI is expected to improve upon this method by selectively enhancing brightness in dark areas of an endoscopic image and subtle tissue differences such as slight morphological or colour changes while simultaneously preventing over-enhancement [12,21,22]. However, the efficacy of TXI for evaluating the condition of the gastric mucosa and severity of gastritis remains unclear. In a small study of patients receiving oral endoscopy (*n* = 12), Ishikawa et al. [23] reported that the colour difference surrounding an atrophic border and gastric cancer border was significantly greater in TXI than in WLI (atrophy: 14.2 ± 8.0 vs. 8.7 ± 4.2, *p* < .01; gastric cancer: 18.7 ± 16.0 vs. 8.0 ± 4.2, *p* < .01). Dobashi et al. [24] reported that Δ*E* values for visibility for squamous cell carcinoma suspicious lesions in the pharynx and oesophagus in TXI mode 1 (texture and brightness, and colour enhancement, 18.6), TXI mode 2 (texture and brightness enhancement, 14.3), and NBI (17.3) were significantly higher than those in WLI (11.6, *p* < .001). Therefore, TXI may be a useful observation modality in endoscopic screening. Our findings provide the first evidence of the efficacy of TXI for detecting intestinal metaplasia as a precancerous lesion, demonstrating that, in addition to greater colour differences surrounding atrophic borders, TXI and NBI also produce significantly larger differences surrounding intestinal metaplasia than WLI. This observation suggests that combining WLI and TXI or NBI in a health check-up may improve detection rates of atrophy and intestinal metaplasia.

Regular endoscopic surveillance increases survival rates of gastric cancer, and >95% of gastric cancer cases identified by annual endoscopy surveillance were cured by endoscopic resection in a Japanese cohort study [25]. Therefore, the importance of endoscopic surveillance has been confirmed in multiple countries [26]. Because transnasal ultrathin endoscopy is safe and can be performed without any sedation, endoscopy is often performed transnasally to reduce invasiveness and distress to the patient [27,28]. However, major disadvantages of transnasal endoscopy include the need for complex considerations (e.g. anaesthesia of the nasal cavity, use of vasoconstrictors, limited manipulations and lower power aspiration and air-supply), poor image quality and lower detection rate of early-stage gastric cancer [29]. However, the image quality and detection rate have improved markedly with the latest ultrathin high-vision endoscopes. In addition, we have demonstrated here that combining NBI or TXI with high-vision endoscopy produces significantly greater colour differences surrounding atrophic borders and intestinal metaplasia than WLI. Therefore, combining TXI or NBI with high-vision transnasal endoscopy may overcome the low detection rates for atrophy and intestinal metaplasia at health check-ups. Such improved screening methods for gastric cancer by transnasal endoscopy will become increasingly important as the number of patients who have received *H. pylori* eradication therapy increases in parallel with the rise in eradication therapy.

This study has a few limitations. First, it was a single-center study. Second, the sample size was small because it was a pilot study. Third, while we examined high-vision transnasal endoscopy images and the new EVIS X-1 processor, we did not directly compare their efficacy for assessing gastritis severity with oral endoscopy, nor did we compare the new processor with older ones. Fourth, we did not compare status of atrophy and intestinal metaplasia between pathological and endoscopic evaluations. In clinical practice, however, it is hard to evaluate status of atrophy and intestinal metaplasia related with gastric cancer risk by pathological evaluation in all patients, due to an increased risk for haemorrhage and cost. Furthermore, endoscopic atrophic border were reported to correlate with pathological evaluations and serum pepsinogen level [30] and the correlation with pathological and endoscopic evaluations for intestinal metaplasia were improved by IEE findings [8]. Therefore, we focussed to compare endoscopic evaluations of atrophy and intestinal metaplasia among WLI, NBI and TXI in this study, not pathological findings. However, because comparison between pathological and endoscopic evaluations (NBI and TXI) is important, we will plan to clarify clinical significance by above comparison, as the further study.

## 5. Conclusion

Given that advances in endoscopic technology have markedly enhanced the diagnostic capability of endoscopy, it is important to identify the best diagnostic method for gastric cancer. Despite sufficient illumination by a new processor for endoscopy, abnormalities in mucosal discolouration and morphological changes to the mucosal surface often go undetected using WLI. In this study, we showed that high-vision transnasal endoscopy with visible colour differences enabled by TXI or NBI is useful and effective for diagnosing atrophy and intestinal metaplasia. Large-scale multi-center prospective studies are needed to investigate the efficacy of TXI and NBI with high-vision transnasal endoscopy and a new processor for detecting atrophy, intestinal metaplasia, and gastric cancer and for identifying patients at high risk of gastric cancer.

## Data Availability

The data based on the results of the current study were obtained, are accessible from the corresponding authors upon reasonable request.

## Abbreviations

*H. pylori*: *Helicobacter pylori*
IEE: image-enhanced endoscopy
IM: intestinal metaplasia
NBI: narrow-band imaging
SD: standard deviation
TXI: texture and colour enhancement imaging
WLI: white-light imaging
